# A Case of Diphtheria Benefited by Inhalation of Steam and Terminating Fatally

**Published:** 1878-03

**Authors:** R. M. Wilson

**Affiliations:** Lincoln, Ill.


*A Case of Diphtheria Benefited by the Inhalation of Steam and Terminating Fatally.*

I do not report the following case because of any peculiarity in its treatment, but to show the advantage that may be derived from the inhalation of steam in the treatment of diphtheria, and to indicate that a case otherwise progressing favorably may suddenly terminate fatally from a cause not reasonably to be anticipated. I refer to death during a favorably progressing convalescence from syncope or paralysis of the heart. I had conducted many cases of this disease to a favorable issue, and had regarded convalescence as a safe indication that the patient was out of immediate danger before the experience here gained. The sudden and unexpected result of this case led me to search the literature of the subject more carefully, and I find but one author who alludes to this source of danger in a manner that would excite apprehension or necessitate a guarded prognosis. Flint, in his work on practical medicine, refers to this source of danger in the following language: "A fact important to be borne in mind is the liability to sudden fatal syncope in this disease. This has occurred unexpectedly in two cases under my observation. It may occur in cases which, as regards the general symptoms, do not present any appearance of great gravity. It generally occurs on some unusual exertion, as in getting out of bed. It has been known to occur during convalescence." Oertel, in his exhaustive article on diphtheria, in Ziemssen's Cyclopædia of Practical Medicine, refers to paralysis of the heart as a cause of sudden death, but does not advise a decided prognosis.

Clifford C——, aged seven years. On September 8th, 1877, this little patient was attacked with soreness of throat and vomiting; pulse 140 per minute; skin dry and pungent. An examination of the throat revealed greatly enlarged tonsils covered with pus, the uvula elongated and œdematous, presenting a glistening appearance. I used spray of carbolic acid to the throat, and ordered a saline purgative and the tincture of aconite root in one drop doses every hour.

Sept. 9th.—Pulse 135; tonsils almost free from pus, but the uvula very much enlarged and completely encased in a diphtheritic exudate. I continued the aconite at longer intervals, and ordered sodic hyposulphite, tincture of iron and potassic chlorate in doses sufficient to bring the system rapidly and completely under their influence.

Sept. 10th.—Pulse 135, and the entire fauces covered with diphtheritic exudate; the tonsils are enlarged till they meet and push the uvula forward on the tongue; discontinued the aconite.

Sept. 11th.—Pulse 135, and feebler.

Sept. 12th.—Pulse 135, denoting asthenia; slight delirium. Continued the treatment of the previous days with the addition of stimulants.

Sept. 13th.—Larynx involved by extension of the inflammation; breathing labored and stridulous; increased the amount of stimulants, and ordered the temperature of the room to be kept as nearly as possible at 80° F., the atmosphere to be kept thoroughly charged with steam. The effect of steam in this case was most gratifying, not failing in a single instance to mitigate the paroxysms of painful and difficult breathing. The air of the room was kept constantly moist with steam, and when the paroxysms of difficult breathing occurred, the steam generator was placed at the bedside so that a dense volume would envelope the head and face. This plan of treatment was continued throughout the next forty-eight hours, during which time his condition remained very much the same.

Sept. 16th.—Patient worse; death from apnœa seemed imminent, but during the day a part of the membrane was dislodged by a violent effort at coughing which afforded great relief in breathing.

Sept. 17th.—Patient better; breathing easy and unobstructed. Prescribed tonics and stimulants in conjunction with nourishing diet.

Sept. 18th.—Patient continues to improve.

Sept. 19th.—Very much better; begs for food.

Sept. 20th.—Vomited during the night from taking too much solid food; prescribed pepsin and hydrochloric acid.

Sept. 21st.—Vomiting checked, and the appetite good. The patient, under a restorative plan of treatment, continued to improve until the evening of the 24th, at which time, upon being lifted out of bed, he instantly turned pale, the surface became icy cold, and he died within six hours.

R. M. WILSON.

LINCOLN, ILL., Jan. 17, 1878.

